# *Conceptual Utility Model for the Management of Stress and Psychological Wellbeing*, CMMSPW^™^ in a university environment: theoretical basis, structure and functionality

**DOI:** 10.3389/fpsyg.2023.1299224

**Published:** 2024-01-31

**Authors:** Jesús de la Fuente, José Manuel Martínez-Vicente

**Affiliations:** ^1^Department of Teoría y Métodos de Investigación Educativa y Psicológica, School of Education and Psychology, University of Navarra, Pamplona, Spain; ^2^Department of Psychology, School of Psychology, University of Almería, Almería, Spain

**Keywords:** conceptual utility model, stress and psychological wellbeing, 3P model, self-regulation vs. external regulation behavior theory, competence model

## Abstract

This article describes and introduces the *Conceptual Utility Model for the Management of Stress and Psychological Wellbeing*, *CMMSPW*^™^ Its purpose is to assess, evaluate and treat stress and psychological wellbeing. First, the *theoretical assumptions* of the model are presented. This model is an application of the 3P Model, Theory of Internal vs. External Behavioral Regulation and the Model of Competency for the Management of Stress and Psychological Wellbeing. Second, the *conceptual structure of the model* is presented. This model allows the structural and functional determination of the variables and predictive, mediating and final factors for stress and psychological wellbeing. Third, the *functional structure* is presented. For predictive factors, the *internal and external self-regulation* theoretical model allows us to assess levels of internal and external regulation of the individual and their context, as well as other personal and contextual factors involved in self-regulation. For mediating factors, the *model of competence* for the management of stress and wellbeing allows us to analyze conceptual (concept and principles), mediating (skills and metaskills) and attitudinal (attitudes, values and habits) variables. Finally, in relation to factors that condition outcomes, we can determine *levels of response to stress and psychological wellbeing*. Finally, *limitations* and *conclusions* are presented. The model also allows us to determine predictive relationships between those three types of variables and is functionally transferable to other contexts, including contexts proper to the psychology of education, clinical practice and healthcare, and psychosocial, organizational and technological contexts.

## 1 Introduction

The creation in an area of study of new conceptual models—with the potential to explain and predict—based on scientific evidence is the proper endeavor of science (in general) and of the Psychological Sciences (in particular). To that end, existing conceptual models are melded with newly created models so as to allow us to better understand and expand on the variability of dependent variables explained by such models. That is the subject of this paper, in relation to research into stress and psychological wellbeing in different contexts.

The *objective* of this study is two-fold: (1) At a *conceptual level*, to present and justify the partial models on which the general conceptual model or heuristic put forward is based, to assist the reader and those using the model to better understand it. (2) In addition, on an *applied level*, the objective includes establishing the utility of the model in interventional assessment processes having to do with stress and wellbeing among university students.

## 2 Justification

The inform research presented has a double justification, both theoretical and applied:

1. At a *theoretical level*, in current psychological science, there is a recognized need to advance towards broader and more integrative conceptual theoretical models, which lead to more efficient explanation and prediction of the role of the numerous variables involved in behavioral variability. That is, a mature level of psychological knowledge enables progress from discrete and specific models, specific to each area of knowledge, towards broader, molar models of an interdisciplinary scope (Mastrokoukou and Crawford-Lee, 2023).
2. Traditionally, explanatory conceptual models in Psychology have been developed in the context of a specific discipline. For example, in analyzing the problem of *stress and well-being* at university, most of the existing models and evidence take a marked neuropsychological, clinical, health-related view (Gu and Mao, 2023; Wong and Yuen, 2023), and do not include the psychoeducational view, connected to the context of teaching-learning processes and other contextual variables. This positioning constitutes a microanalysis or molecular-clinical focus of analysis, ignoring the contextual-molar or interactive level (de la Fuente et al., 2019). However, in many cases they have not been extrapolated to other contexts due to the theoretical and empirical difficulty of validation in different contexts. In practice, that has made it much harder to generalize psychological theories, given that the majority of models have been restricted to the specific *theoretical domain* or *knowledge area* in which they arose. For that reason, it has generally been difficult to test explanatory mechanisms for specific problems in other academic or professional fields. There are a number of exceptions in relation to general models and theories of motivation and personality. In this case, the present conceptual model takes an *omnibus-model* view, and can be used in the spheres of educational psychology, clinical and health psychology, and organizational psychology (de la Fuente and Martínez-Vicente, 2023b,c).
3. At the *applied level,* the contribution of new, evidence-based conceptual utility models represents a professional innovation of the first order. New tools or heuristics for analysis, evaluation and applied professional decision-making become possible. In the field of innovation, there are differences between a patent and a utility model. Patents protect the invention of something that is completely new (such as vaccines against COVID-19), while utility models incorporate a useful improvement of something that already existed. The patent and the utility model are titles granted by the State and give their holder the right to temporarily prevent others from manufacturing, selling or commercially using the protected invention in a given country. Term of ownership is twenty years from the filing date in the case of patents and ten years for utility models. Once the duration has elapsed, the invention is in the public domain and anyone can use it freely (Ministry of Industry, Energy and Commerce, 2024).

## 3 Theoretical basis

### 3.1 Foundational models that precede the new conceptual utility model

#### 3.1.1 Reasons for a new model

The proposed utility model aims to address an unresolved need in previous stress models, which have the following characteristics:

1. They take the conceptual view of stress as a maladaptive response, and give priority to a biological approach (Gulewitsch et al., 2013; Godoy et al., 2018) to the detriment of psycho-social factors of stress. If we wish to adopt a more balanced bio-psycho-social paradigm (WHO, 2001), models must be developed that adequately integrate psychological and contextual factors, due to their functional, predisposing value in explaining stress.
2. They assume that stress is an essentially individual problem, derived from the subject’s personality. For this reason, they focus on molecular explanatory mechanisms or the subjects themselves (Pozos-Radillo et al., 2014, 2015; Amanvermez et al., 2020), to the detriment of contextual factors, specific to the educational context. They do not adopt an interactive view, which is key to a better understanding of the phenomenon of academic stress.
3. They take into consideration predictive variables in the subject as determinants of the level of stress (Restrepo et al., 2023), but do not sufficiently incorporate mediating variables, namely, the subject’s level of competence, which constitutes a protective factor, stress inhibitor and promoter of well-being. Such variables serve to minimize stress responses and maximize the subjects’ well-being.
4. A large number of models are focused on the negative pole of the behavioral continuum. Thus, they aim to analyze the predictive and constitutive factors of stress responses (Hoge et al., 2023). However, the positive pole or behavior that promotes well-being is not defined in the same terms.

#### 3.1.2 Advances of the new model

The proposed Conceptual Utility Model (de la Fuente et al., 2022a,b,c; de la Fuente and Martínez-Vicente, 2023a,b,c) aims to address and overcome the above limitations in an integrative heuristic based on prior evidence (de la Fuente, 2021). It seeks to provide a general model applicable in different psychological fields, and to be both protective and predictive of stress and psychological wellbeing:

1. In terms of *presage* var*iables*, this model starts from the 3P model (Biggs, 1999) which affirms the existence of presage (predictive) variables, process (mediating) variables and product (dependent) variables. To complement the 3P model in terms of presage variables, *the Self- vs External-Regulation Behavior Theory* model (de la Fuente et al., 2017, 2021a,b; de la Fuente et al., 2022a,b,c) has proposed *Regulatory/Non-Regulatory/Dysregulatory* levels for the individual and the context, based on biomedical models of dysregulation (Shields et al., 2017).
2. In terms of *process* var*iables*, the 3P model has been complemented by the personal competence model (Gagné, 1965; de la Fuente et al., 2018a,b). This conceptual model has established different types of learning that a human being must present in order to be competent in the management of *stress and psychological wellbeing,* namely: (1) conceptual; (2) procedural; (3) attitudinal.
3. In terms of product or predictive *final variables*, we have incorporated the model of *experience* of academic stress (Stallman, 2010; de la Fuente et al., 2015a) and *psychological wellbeing* (Ryff and Keyes, 1995; Ryff and Singer, 1996).

### 3.2 Foundational conceptual model underlying the utility model

The proposed heuristic, *Conceptual Model for the Management of Stress and Psychological Wellbeing*, *CMMSPW*™, integrates and synthesizes prior conceptual models.

#### 3.2.1 Biggs’ 3P model

**Figure 1:**
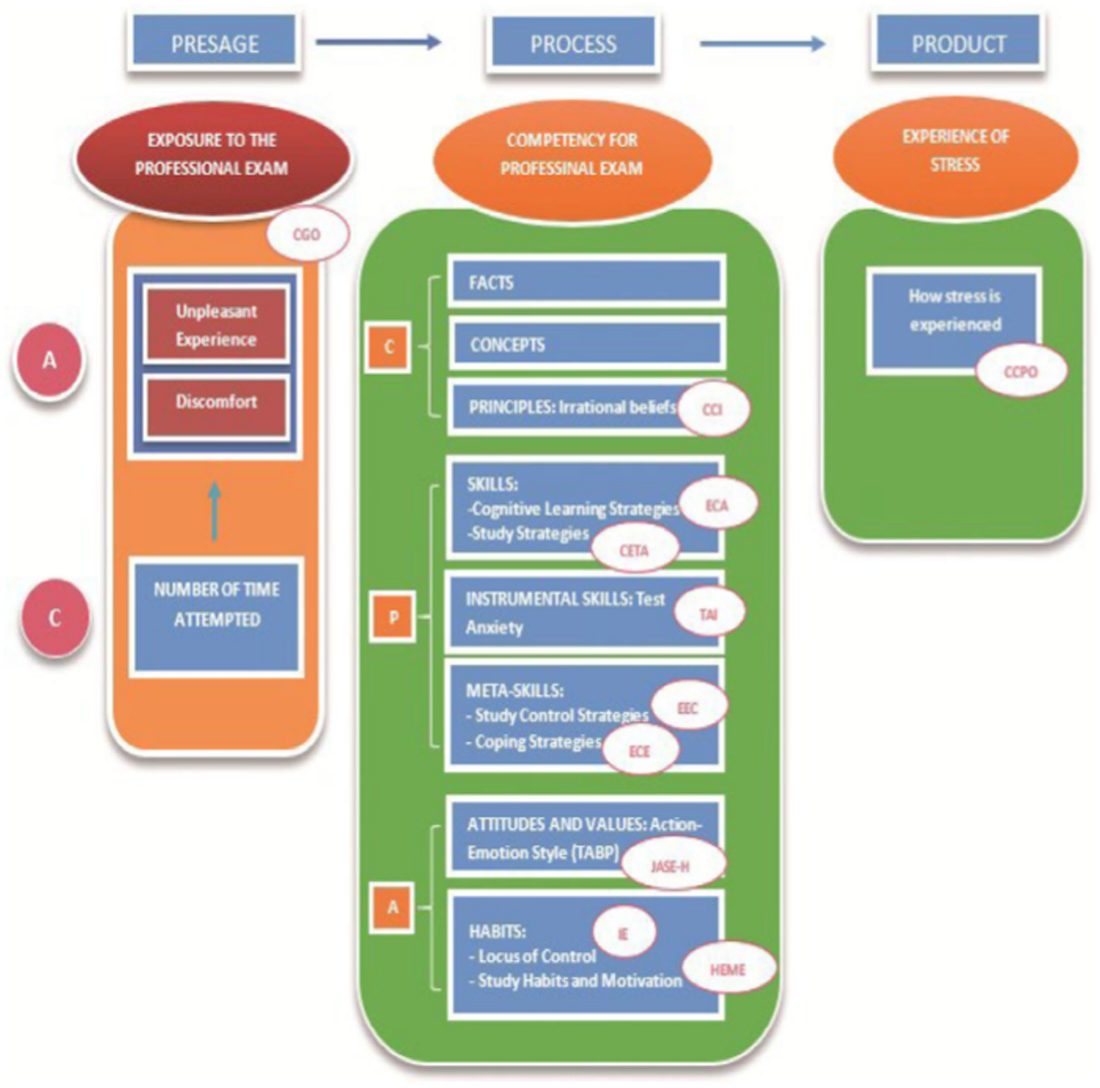
Competency model for studying, learning and performing under stress (de la Fuente et al., 2015a,b,c), showing variables and some of the assessment instruments used. Reproduced with permission.

The Ps in the name of this model stand for Presage-Process-Product (Biggs, 1993, 1999). As a sequential model, it is a good representation of academic reality at university and enables us to understand and assess the factors inherent to university learning. It has generated copious evidence (Zhang, 2000; Zeegers, 2001; Rosário et al., 2005; Sarzoza, 2023) and continues to do so (Yang and Lin, 2023) (see Figure 1).

A strength of the 3P model is that it allows us to determine probabilistic relationships within the model among various significant variables that may be predictive of and mediate the ultimate variable of academic performance:

1. In terms of *predictive factors* (presage), it identifies as factors that predict a student’s learning style:
a. The learner’s *individual characteristics*, such as age, gender (Cano-García, 2000; Cano-García and García-Berbén, 2009; de la Fuente et al., 2013), expectations of self-efficacy (Prat-Sala and Redford, 2010), notions about learning (Richardson, 2011), personality traits (de la Fuente et al., 2020a,b,c,d), as predictive and causal factors of university learning. It also determines relationships involving the self-regulatory traits of students and their learning focus (Heikkilä and Lonka, 2006; de la Fuente et al., 2008; Rosário et al., 2010).
b. Characteristics of the *context* in which learning takes place, such as the nature of the institution (Bliuc et al., 2011) and the nature of the course content and teaching methods (Trigwell and Prosser, 1991; Rosário et al., 2014) as factors that are propitious for university learning. Initially, in the study of the stress and well-being model, this variable was not considered. Subsequently, the importance of introducing this category of variables was confirmed.
2. In terms of *process factors*, the model initially focused on the analysis of individual learning factors, to the detriment of contextual factors:
a. The *learner’s individual characteristics* the model identifies as factors likely or probable to varying degrees to mediate the process, students’ habitual study methods (Thompson and Lake, 2023). And the student’s motivation and study strategies (Valle-Arias et al., 1998; Cano-García and Hughes, 2000). Alongside that, learning focuses have been compared with learning styles, with consistent results (Gargallo-López et al., 2013).
b. The initial model did not consider characteristics of the context or the interaction between teaching and learning to explain the type of cognitive, motivational and behavioral strategies during learning.
3. Finally, and in terms of *product or outcome factors*, academic performance and satisfaction with the learning process (Zapata, 2013) are essential variables. In this case, relationships were established between self-regulatory characteristics and the focuses of self-regulation in learning with the type of performance (de la Fuente et al., 2008). Also in specific areas of learning (Cano-García et al., 2014).

#### 3.2.2 The DEDEPRO model

**Figure 2:**
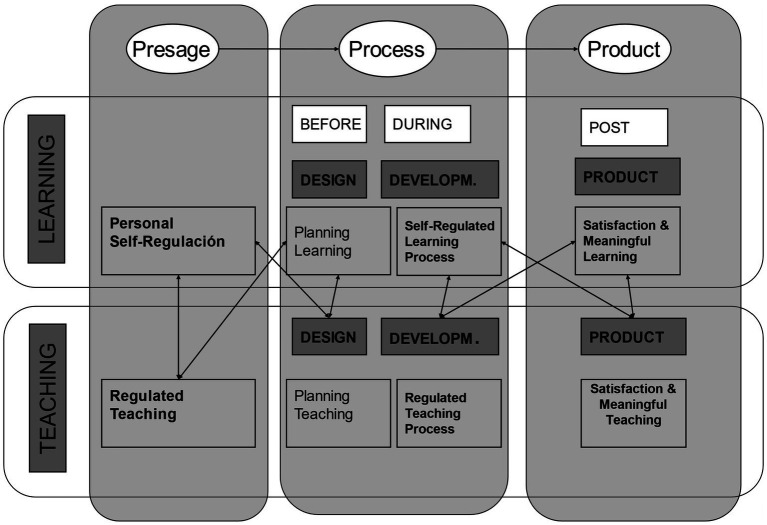
The DIDEPRO model, in the context of 3P Model and the teaching and learning models. Reproduced with permission.

The 3P model was subsequently improved and completed in terms of process or mediating factors in the form of the Design-Development-Product, DE-DE-PRO (from the initial letters of the Spanish words) conceptual model (de la Fuente et al., 2006, 2011; de la Fuente, 2011), in the field of university education to provide greater explicitness about factors that affect design, implementation and outcome of the *teaching-learning process* in a university context (de la Fuente et al., 2006, 2014a,b; Cheng, 2022). Although the original 3P model (Biggs, 1999) implicitly identified variables involved in teaching and learning, it did not provide an exhaustive or explicit description of the possible relationships among the variables in the original model. In fact, the model helped to define the interaction among those variables (see Figure 2):

1. In terms of *presage*, it identified as *predictive factors* learning style, (1) the *personality* of the individual learner, and their age, sex and personality type and (2) the characteristics of the *context of learning*, such as the type of institution, course content and methods of delivery and effective teaching, in terms of the way in which course content and delivery regulates teaching and learning (de la Fuente et al., 2011).
2. In terms of *process*, it identified as *mediating factors* the habitual learning style or *learning focus* of each student (Karagiannopoulou et al., 2020; Xie et al., 2022). And the motivation and learning strategy of the individual (Dinsmore et al., 2020) and *effective teaching*, in terms of the way that course delivery regulates teaching and learning (de la Fuente et al., 2011, 2016b).
3. Finally, and in terms of *product or outcome factors*, academic performance and satisfaction with the learning process (Littman-Ovadia and Freidlin, 2022).

#### 3.2.3 The internal and external regulation of learning SRL-ERL model

As a third stage in this process, the *Self- vs External Behavior Learning Theory*, SRL-ERL (de la Fuente et al., 2017) was put forward to explain the different types of interaction between types of *self-regulated learning* (Regulated/Unregulated/Dysregulated) and *regulatory teaching* (Regulatory/Non-regulatory/Dysregulatory). It arises in the psychology of education to create a heuristic capable of making specific predictions concerning the combination of the degree of regulation of learning by a student and by the teaching process in terms of how that combination affects academic performance (de la Fuente et al., 2012a,b).

Against that theoretical background, in a similar way to metacognitive variables intrinsic to self-regulated learning (Zimmerman and Martinez-Pons, 1986; Zimmerman, 1990, 1998, 2000; Zimmerman and Risemberg, 1997; Zimmerman and Schunk, 2001; Moohr et al., 2021; Zachariou and Whitebread, 2022), which have generated a large volume of evidence concerning their impact on learning, we have postulated the existence of different levels of regulation in students: regulation/non-regulation/dysregulation (SR-NR-DR).

Having examined the role of effective teaching practice, we also postulated equivalent levels for teaching: external regulatory/external non-regulatory/external dysregulatory (ER-ENR-EDR). The empirical confirmation of the theoretical and empirical significance of those three combined levels produced large amounts of evidence (de la Fuente et al., 2017, 2019). That in turn led us to formulate the theory of internal and external regulation of learning, the *SRL vs ERL Theory* (de la Fuente et al., 2017).

Following confirmation of the correspondence between theory and data in that area, we started to test the importance of personal and contextual factors of stress and psychological wellbeing in other contexts. Considerable evidence led to the conclusion that the variability of many recent research variables are predicted and determined by the combination of levels of internal and external regulation. That is the case for resilience, academic emotions, degree of procrastination, levels of stress and academic performance itself (de la Fuente et al., 2017, 2018a,b, 2019). We finally put forward an integrated predictive model, with protective and risk factors for academic stress relating to the individual and their context (de la Fuente et al., 2021a,b).

#### 3.2.4 The self-regulatory vs external regulatory behavior theory

However, that model was very specific and was created specifically for the field of the psychology of education. Having shown that it accurately modeled the phenomena addressed, it was decided to extrapolate the model to other contexts. That led to the need to devise a theoretical model that adequately determined the *person x context* interaction in general terms in different contexts.

From that starting point, the new model sought to extrapolate the specific model from the field of education to other psychological contexts, leading to the model in *Self- vs External-Regulation Behavior Theory* (de la Fuente et al., 2021a,b, 2022a,b,c), as a *general model of regulatory behavior* that could apply to different fields: Psychology of education and ICT, Clinical and Health Psychology, Social and Organizational Psychology, and other contexts (de la Fuente et al., 2016a, 2022a,b,c). To that end, we created and validated specific evaluation tools for use in the different fields (de la Fuente et al., 2022a,b,c; de la Fuente, 2024a,b).

**Figure 3:**
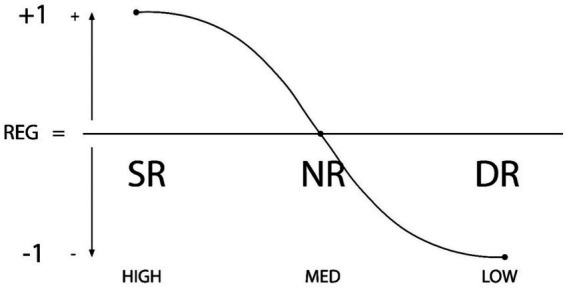
Graphic representation of individual regulation types: SR (Self-regulation), NR (Non-regulation or De-Regulation) and DR (Dys-Regulation). The X axis represents the degree of regulation (high-medium-low), while the Y axis shows directionality (+1, 0, −1). Reproduced with permission (de la Fuente et al., 2022a,b,c, p. 17).

**Figure 4:**
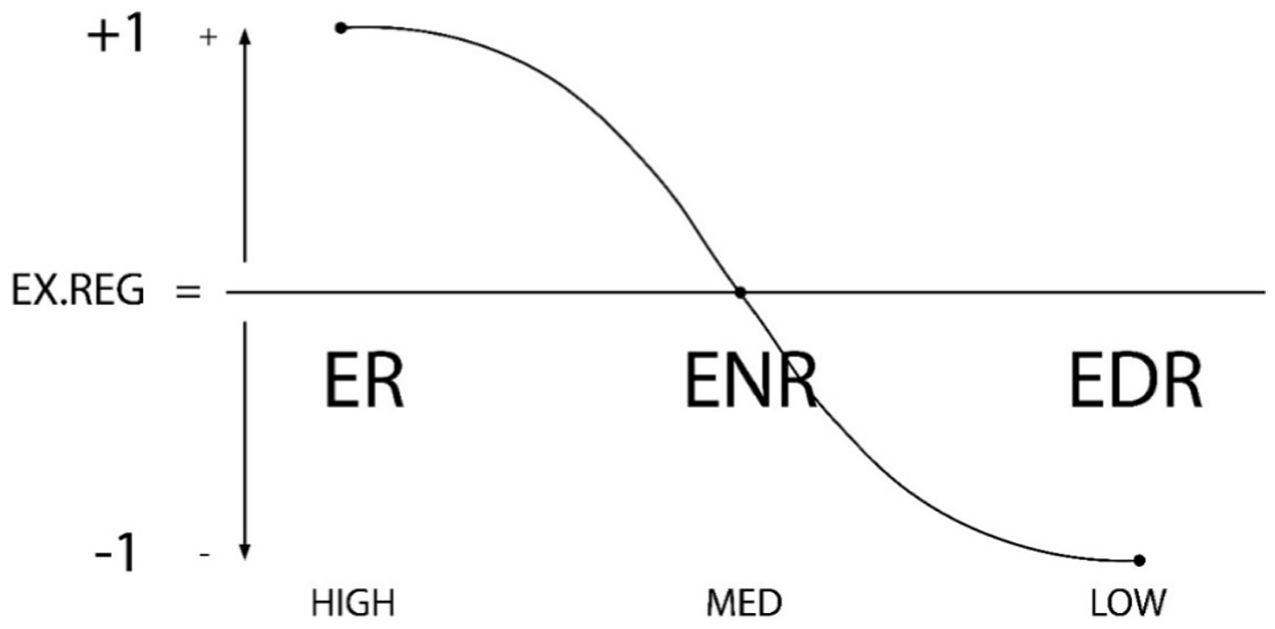
Graphic representation of external or contextual regulation types: ER (External Regulation), ENR (External Non-regulation or De-regulation) and EDR (External Dys-Regulation). The X axis represents the degree of external regulation (high-medium-low), while the Y axis shows the directionality of the external regulation (+1, 0, −1). Reproduced with permission (de la Fuente et al., 2022a,b,c, p. 17).

Thus the significance of this new—more general—model is that it allows the identification and assessment of personal and contextual regulation as a predictive (presage) variable for purposes of psychological assessment and treatment in the fields mentioned (see Figures 3, 4).

#### 3.2.5 Competence for human learning

Since Gagné (1965) introduced his *instructional model of teaching and learning* of differential learning which allows a human being to be competent in a given field of learning and development, that model has been extrapolated to other areas. This comprehensive holistic model allows us to integrate partial contributions from other cognitive-behavioral models of stress and other issues. Thus, researchers have described competence to interact with alcohol (de la Fuente et al., 2017) and competence in avoiding and dealing with academic stress (de la Fuente et al., 2015a,b,c).

Evidence has emerged from our field of investigation of the relationships between different levels of variables inherent to competency. In essence, the model summarizes the levels of learning that a person needs to have in a given domain: KNOWLEDGE (FAMILIARITY) + KNOW-HOW (ABILITY) + KNOWING HOW TO BE (WANTING). However, this schematic or heuristic, despite its power as a tool to bring together different strands of research, has not been taken up in full by different fields in psychology to assess and intervene in relation to the competencies of individuals in connection with a given behavioral problem (see Table 1).

**Table 1:** Structure of learning of competencies (de la Fuente et al., 2015a), based on R. Gagné (1965).

KNOWLEDGE (Knowledge)	Knowledge of facts about the learning domain
	Familiarity with concepts concerning the domain
	Knowledge of principles concerning a domain
KNOW-HOW: (Capacity)	Self-management skills in a given behavioral domain
	Self-management metaskills in the relevant behavioral domain
KNOWING HOW TO BE: (Wanting)	Attitudes particular to a domain
	Values particular to a domain
	Habits particular to a domain

1.In the case of *stress-management competence*, a person is said to be competent to manage stress when they present with three levels of behavior referred to above, to adequately manage stress situations in different settings: academic, health, personal.
2.In a similar way, in the case of *competence for the management of psychological wellbeing*, a person is said to be competent to achieve a state of psychological wellbeing when they present the three levels of behavior referred to above, to adequately manage experiences and states of psychological wellbeing in different situations (de la Fuente et al., 2022a,b,c).

#### 3.2.6 Model of stress and psychological wellbeing

The assumed *model of stress* arising from negative psychology or psychopathology based on individual risk factors is particular to the responses that constitute and correlate with stress (Stallman, 2010; de la Fuente et al., 2012a).

The assumed *model of psychological wellbeing* which arises from positive psychology, based on individual protective factors is a combination of hedonic models, which focus on the prevalence of emotionally positive wellbeing (Diener et al., 1985; Disabato et al., 2016) and eudaimonic models which focus on the prevalence of teleological wellbeing (Ryff and Keyes, 1995; Ryff and Singer, 1996).

## 4 Structure: *conceptual utility model for Management of Stress and Psychological Wellbeing*, CMMSPW^™^ in different settings

The proposed utility model (de la Fuente and Martínez-Vicente, 2004, 2023a,b) is an integrative heuristic based on prior evidence (de la Fuente, 2021). It seeks to provide a general model applicable in different psychological fields, and to be both protective and predictive of stress and psychological wellbeing (See Supplementary Appendix S1). In this previous empirical synthesis work, a joint structural predictive model of personal and contextual factors that significantly probabilize a final experience of well-being or psychological stress was shown. It reflects, structurally, individual and contextual factors, which have served as structural support for the current utility model.

This article reflects the specific structural variables used in the *psychoeducational context* (see Supplementary Appendix S2):

1. In terms of *presage variables*, this model affirms that presage or distal predictive variables can be individual or contextual:
*Individual variables*: based on the results of previous research, the variables of students’ age and gender, personality (Big Five), positive and negative affect, and level of regulation were considered in the model. Taking the perspective of the Theory of Self- vs. External-Regulation Behavior (de la Fuente et al., 2017, 2021a,b, 2022a,b,c), the model distinguishes Regulatory/Non-Regulatory/Dysregulatory levels for the individual, based on biomedical models of dysregulation (Shields et al., 2017).
*Contextual* var*iables*: the level of external contextual regulation has also been identified by the Self- vs. External-Regulation Behavior Theory (de la Fuente et al., 2017, 2021a,b, 2022a,b,c; Pachón-Basallo et al., 2022): externally regulatory / external non-regulation/externally dys-regulatory. Additionally, the family support variable has been taken into consideration in the educational context, due to its great relevance.
2. In terms of *process or mediating variables*, the model includes two levels of variables that previous research has shown to be very relevant:

*Individual variables.* This conceptual model claims that a human being must acquire different types of learning in order to be competent in managing their stress and psychological well-being (de la Fuente, 2023a), namely: (1) conceptual; (2) procedural; (3) attitudinal. These three levels are essential to a self-regulatory, meaningful learning process (de la Fuente and Eissa, 2023), and to the competence of managing stress and well-being, especially at the level of meta-skills (de la Fuente et al., 2023b), as will be explained in the next section.
*Contextual* var*iables.* An important contribution of this conceptual model is the integration of teaching processes, as contextual factors that may promote stress responses and that mediate the students’ state of stress or well-being (de la Fuente et al., 2015a). This contribution has been possible thanks to the continued study of academic stress, in the context of teaching-learning processes (de la Fuente et al., 2023a,b).

3. *Product* or *predictive final* var*iables*. The model has focused its attention on the final experience of the subjects:
*Individual variables*: we have incorporated students’ *experience* of *academic stress* (Stallman, 2010; de la Fuente et al., 2015a) and their *psychological wellbeing* (Ryff and Keyes, 1995; Ryff and Singer, 1996).

In summary, the structure proposed in the new conceptual utility model makes it possible to work at two levels:

1. A *multidimensional structure* at the molar level (de la Fuente et al., 2021a), which furthers multidirectional and interactive analysis, building on the partial proposals of previous models at the molecular level (3P, SR-ER model, DIDEPRO or Competence models).
2. A *multidisciplinary structure*, addressing stress and psychological well-being across different areas of psychology. As has been noted, this manuscript presents only the relationships in the field of educational psychology. Current research is analyzing the model’s empirical functioning in the different areas it addresses: educational psychology, health-related psychology and organizational psychology. Future research will determine, based on evidence, whether the model presented is sufficiently robust in its current form.

## 5 Functionality: the conceptual model as a heuristic for professional decision-making in different settings

Based on the heuristic or the Utility Conceptual Model^™^ (see Supplementary Appendix S1), we have proposed the assessment and improvement of specific variables, applying in each context the variables that evidence has shown to be essential (de la Fuente and Martínez-Vicente, 2023b,c). Here, we provide an explanation-synthesis of these variables only in the context of educational psychology (see Supplementary Appendix S2).

### 5.1 Functional analysis based on the heuristic in the sphere of the educational psychology at university

#### 5.1.1 General functionality

The main contribution of the conceptual utility model is that it provides a general conceptual map (see Supplementary Appendix S1) and other specific maps according to area (see Supplementary Appendix S2). These allow the psychologist to identify, evaluate and intervene in the variables established therein (see the full proposal: de la Fuente and Martínez-Vicente, 2023b,c). It is thus possible to:

1. Conceptualize and test the hypothesized relationships, and so provide empirical evidence of such relationships in a given study population: students, patients, workers. An example of recent research contributions and research in progress can be found on the Project website: https://www.inetas.net/stress/seccion.php?ididioma=1&idseccion=6&idproyecto=10
2. Conceptualize and carry out explanatory predictive hypotheses, in an analysis of a given case, to make an assessment and subsequently intervene in the selected variables.

#### 5.1.2 Specific functionality in the educational psychology context

Based on the 3P model (Biggs, 1999), noted above, the heuristic has selected variables on the basis of ample prior evidence that are of significance to this field of investigation:

##### 5.1.2.1 Presage (predictive) variables

###### 5.1.2.1.1 Personal presage variables

The age and sex of each individual student have been seen to be relevant differentially predictive factors of learning behaviors (Weis et al., 2013; Rubin et al., 2018; López-Madrigal et al., 2021; Rubach et al., 2022; de la Fuente et al., 2023a). As such they are important to the determination of cognitive and emotional differences among students in learning processes.

Another individual variable that research has shown to be relevant is *Personality*, specifically the Big Five model, as a distinctive personal characteristic of students (Poropat, 2009; Backmann et al., 2019; Sander and de la Fuente, 2022; Spielmann et al., 2022). This predictive factor has appeared as a significant variable in the prediction of cognitive-emotional characteristics of learning: conscientiousness has been shown repeatedly to be associated with and positively predictive of better performance and better strategic learning, whilst neuroticism (lack of emotional stability) is negatively predictive. Now, some works have proposed a sliding scale in personality traits depending on how pro-regulatory each trait is (de la Fuente and Martínez-Vicente, 2004).

Self-regulation as a personality trait among students has also shown itself to be predictive and causative of adaptive vs. non-adaptive behavior in the course of learning (Matthews et al., 2000). There is very extensive evidence of its value in the prediction of the performance of learning behavior by students. The positive association and predictive relationship between self-regulation and subsequent learning behaviors is very consistent (Umerenkova et al., 2022). In fact, it has been found to be predictive of deep, meaningful learning processes (de la Fuente et al., 2015a), and to be predictive of emotional maladjustment in learning (Moohr et al., 2021). Hence the importance of assessing and improving self-regulation (Bittner et al., 2022). In complementary manner, a clear relationship has emerged between self-efficacy and self-regulation (Lin et al., 2023).

More recently, the concept of types of internal and external self-regulation (Self-Regulation/Self-non-regulation/Self-dysregulation SR-NR-DR) has helped to distinguish the types and levels of self-regulatory behaviors in students. Recent evidence has been very consistent in relation to its association with, and linear prediction and determination of, learning focuses amongst students and of other strategic aspects and learning metaskills (de la Fuente et al., 2017, 2022a,b,c).

###### 5.1.2.1.2 Contextual presage variables

The construct *internal or external regulation* (ER/ENR/EDR) has helped to order the desirable and undesirable regulatory effects of students’ contexts. Evidence provided by this construct has shown the importance of regulatory versus non-regulatory and dysregulatory educational or teaching contexts to different learning behaviors during the learning process. They can be identified as protective or risk factors in the learning process (de la Fuente et al., 2021a,b, 2022a,b,c). The *general design of education* has been seen to be a predictive factor (de la Fuente et al., 2020a). Family context has also been shown to be an essential component of context, with a clear role in promoting and facilitating or interfering in processes of motivation and learning (Ross and Hill, 2000; Tapia et al., 2013; Núñez et al., 2015; Boncquet et al., 2022).

##### 5.1.2.2 Process (mediating) variables

###### 5.1.2.2.1 Personal process variables

####### 5.1.2.2.1.1 Conceptual variables (knowledge: concepts)

Learning focus has been shown to be an essential variable to understand cognitive-motivational beliefs and underlying strategies in the course of learning (Shum et al., 2021). With extensive evidence, the model allows us to distinguish academic learning focuses that are more or less adaptive (Heikkilä, 2011; Karagiannopoulou et al., 2018; Panadero et al., 2021; Asikainen et al., 2022).

Alongside that, the variable learning styles also significantly assists us to understand conceptualizations, beliefs and actions concerning academic learning, because that variable tells us about elaborative processing and conceptualizations of the learning process (Entwistle and Ramsden, 1983; Cassidy, 2004; Gargallo-López et al., 2013; Martínez-Fernández and Vermunt, 2015; Vermunt and Donche, 2017).

####### 5.1.2.2.1.2 Procedural variables (know-how)

Skills applied in the learning process have been shown to be essential instrumental elements for adequate learning in an academic context. Skills such as oral expression, note-taking, study techniques and teamwork have been seen to be *basic learning tools* (Sewell et al., 2022). Although they make a relatively small contribution to regulation, they are essential first-order tools in school and university learning. And for that reason, they should be assessed and improved.

At the level of *metaskills or skills in management and regulation of instrumental skills* (de la Fuente et al., 2015a), recent research has generated a large volume of evidence concerning these higher order or *strategic metacognitive* skills in academic learning (Cano-García and Justicia, 1993; Basu and Dixit, 2021; Cai et al., 2022; Krieger et al., 2022; Küçükaydın, 2023; Paz-Baruch and Hazema, 2023). Thus, there have been added to traditional—mostly cognitive—learning strategies, regulatory strategies for the regulation of motivational-affective processes, in other words: *metamotivational and meta-affective strategies.*

1. *Resilience* has been seen as a factor in metamotivational regulation (Grossman, 2014; Artuch-Garde et al., 2017; Dray et al., 2017).
2. *Coping strategies* as a factor in meta-affective management (Banerjee et al., 2019; Freire et al., 2020; de la Fuente et al., 2021b).
3. Self-regulation as a factor in behavioral metaregulation (Blair and Raver, 2015; de la Fuente et al., 2015b).

Research is also providing evidence concerning the pernicious effects of the absence or dysfunction of those skills. Such is the case (4) of *procrastination* as an example of regulatory failure or dysregulation (Garzón-Umerenkova et al., 2018; Netzer-Turgeman and Yehuda Pollak, 2023) and *emotional dysregulation* as difficulty in emotional control (Coifman and Aurora, 2022; de la Fuente et al., 2022b).

####### 5.1.2.2.1.3 Attitudinal variables

*Achievement emotions* are an attitudinal variable which has been shown by copious evidence to be predictive of learning, positive or negative learning experience and final achievement (Reindl et al., 2020; de la Fuente et al., 2020c; Pekrun et al., 2023; Wang and Zheng, 2023). In association with those emotions, academic confidence has emerged as a first-order attitudinal factor which is predictive of learning focus, satisfaction and achievement (de la Fuente et al., 2013; Sander and de la Fuente, 2022; Lu and Wen, 2023).

Action-emotion style has consistently been shown to be predictive and discriminating in relation to learning focuses, emotions, coping strategies and work habits (de la Fuente et al., 2008, 2016c).

Maladaptive perfectionism has emerged as an important mediating factor that modulates motivation and emotional dysregulation in learning (Hill et al., 2020; Lee and Anderman, 2020; Moreno-Peral et al., 2020; Zeifman et al., 2020; de la Fuente et al., 2022c; Sepiadou and Metallidou, 2022; Kahn et al., 2023). On the other hand, adaptive perfectionism correlates with self-expectation and adaptive improvement in different contexts (Flett and Hewitt, 2020).

Personal strengths have emerged as essential (attitudinal) learning variables that comprise numerous emotional-affective skills to undertake the effort required by ongoing university education (Villacís et al., 2021; de la Fuente et al., 2022a).

###### 5.1.2.2.2 Contextual process variables

The effectiveness of the teaching process has proved to be functionally protective against stress by promoting a deep learning approach, learning strategies, problem-focused coping strategies, positive emotionality and, finally, satisfaction with the teaching-learning process, hence, less stress and more well-being (de la Fuente et al., 2021a,b). Previous research has also shown this functional predictive directionality (Mastrokoukou et al., 2022).

##### 5.1.2.3 Product (outcome) variables

*Academic performance*, in the sense of not just an average grade but of the acquisition of skills as applied to a given field of knowledge and practice. This dependent variable has—for obvious reasons—been examined by many researchers (de la Fuente et al., 2010; Barattucci et al., 2021; Casiraghi et al., 2022). Some models have assumed that *academic performance* entails the acquisition of learning or conceptual, procedural and attitudinal subcompetencies in an integrated way (de la Fuente et al., 2005).

*Academic satisfaction* has also been much studied and is considered to be a final or outcome variable, at least as important as academic performance (if not more so) as a correlate of experiences of wellbeing (de la Fuente et al., 2015a).

*Academic stress* has also been seen as a variable, that is predicted by groups of many of the variables previously described. It has been shown to be negatively correlated with experience of satisfaction (de la Fuente, 2021).

*Flourishing, academic health and psychological wellbeing* have been seen as process outcome dependent variables of great contemporary importance (Garzón-Umerenkova et al., 2018; de la Fuente et al., 2022a).

## 6 Applicability of the conceptual utility model: psychoeducational assessment and intervention

The model is being applied in two aspects:

1. This new utility model is guiding the work of our current Knowledge Promotion R&D Project (see Project reference) and will serve to open future avenues of research. Conceptual and predictive relationships inherent to the model have been empirically tested, to determine the precise directionality of the relationships. The model has been partially validated by the preliminary evidence (de la Fuente et al., 2021a,b; see previous sections).
2. Complementarily, an *online self-help tool* has been developed for professional use (see Proof of Concept Project). We consider this an example of how the R&D&I value chain in Psychology can make relevant contributions to the profession (de la Fuente et al., 2018a).

### 6.1 Assessment of each variable in the model

However, this conceptual utility model (de la Fuente and Martínez-Vicente, 2004, 2023a,b,c) allows us to formulate *precise assessment and intervention hypotheses* to support decision-making in professional contexts concerning the psychology of university education. It is a powerful conceptual tool for decision-making in the field of University Guidance supported by the *e-Self-Help Tool, e-Coping with Academic Stress* (de la Fuente et al., 2015c). This model has already been used with educational psychologists for training in assessment, through case studies, in the 2023 academic year. In the same program, based on the real-case approach, variables have been identified and pertinent assessment instruments have been proposed (See Supplementary Appendix S3).

### 6.2 Evidence-based intervention for each variable in the model

Along the lines of evidence-based psycho-educational intervention (Slavin, 2017, 2019; de la Fuente et al., 2023a), proposed interventions and improvement measures have been put forward as strategies for external assistance to improve the specific behavioral variables analyzed.

On the basis of empirical evidence concerning the variables analyzed in the model, the *e-Self-Help Tool, e-Coping with Academic Stress* (de la Fuente et al., 2015c) suggests actions for progressive improvement to address each subcompetency in question. The intervention proposal has been made through the self-help tool or the behavior improvement proposal, through training activities for the subjects (de la Fuente, 2024a).

## 7 Limitations

The *conceptual utility model* presented here has limitations that must be mentioned. Firstly, although it represents a conceptual and empirical advance with respect to the previous models mentioned, and has an omnibus nature, applicable to different fields of psychology, it does not integrate all the possible variables in the areas of stress and psychological well-being. The variables included are very representative, typical of our lines of research. This means that present or future research should continue to incorporate other variables.

Secondly, this model has not yet integrated—although it has the potential to do so—all the relevant recent evidence on the role of emotion regulation variables (Milenios et al., 2021). One future line of work should be precisely the integration of the plentiful, varied evidence, integrating it into the utility model.

Finally, the model has an important limitation referring to the samples used in defining the proposed empirical relationships. The large proportion of university students requires that, in the near future, these analyzes and relationships be tested with other educational, health-related, and organizational samples outside the university environment.

## 8 Conclusion

Evidence-based conceptual utility models—such as the model put forward in this report—should be seen as first-order tools for the transfer of scientific knowledge to the field of applied psychology. They represent in themselves a significant advance in knowledge of the Psychological of Education and they allow:

1. The identification of complex problems on the basis of prior research and the construction of hypotheses that are explanatory and predictive of those problems. That is an essential professional competence for those working in the psychology of education. These models allow account to be taken of predictive and risk factors for university students and their contexts (de la Fuente, 2021).
2. The deductive identification of factors or variables to be assessed, associated with assessment instruments (translated and validated) tested in the population in which they are to be used. That represents an unequivocal advantage, in light of the research tools that the model brings to research in the psychology of education that have originated in the Anglosphere, such that they must be adapted for use in other cultural contexts.
3. The putting forward of discrete interventions, based on the direction determined by evidence and adjusted to each variable under analysis (de la Fuente et al., 2023a,b).

In summary, this model allows the three essential stages of any professional psychological intervention to be brought together: (1) Explanatory determination of the problem; (2) Assessment and diagnosis of the problem; (3) Intervention using specific techniques and actions. That competence is included in international professional standards (EuroPsych, 2022).

It also contributes to the R&D&I value chain (Research + Development + Innovation) through specific models of wide professional application in the practice of the Psychology of Education (de la Fuente et al., 2012a, 2018a). Specifically, this conceptual model has served to support the e-Coping Tool for Academic Stress (de la Fuente, 2023b).

## Data availability statement

The original contributions presented in the study are included in the article/Supplementary materials, further inquiries can be directed to the corresponding author.

## Author contributions

JF: Conceptualization, Funding acquisition, Project administration, Writing – original draft, Writing – review & editing. JM-V: Conceptualization, Formal analysis, Supervision, Writing – review & editing.
