# Supplementary material for: Conceptual Utility Model for the Management of Stress and Psychological Wellbeing, CMMSPW™ in a university environment: theoretical basis, structure and functionality
Source: Front Psychol. 2024 Jan 31;14:1299224. doi: 10.3389/fpsyg.2023.1299224 (PMC10866042; doi:10.3389/fpsyg.2023.1299224)
Supplement: Supplementary file 1 [file Data_Sheet_1.pdf]

ANNEX 1

GENERAL CONCEPTUAL MODEL OF COMPETENCE IN THE MANAGEMENT OF ACADEMIC STRESS AND PSYCHOLOGICAL WELLBEING (de la Fuente, & Martínez-Vicente, 20122; IPR. No 00765-01162097)

PRESAGE V. →	PROCESS V. (mediators) →	PRODUCT V.
PERSONAL	PERSONAL	PERSONAL
**INDIVIDUAL**	**COMPETENCE IN MANAGEMENT STRESS AND WELLBEING**	**WELLBEING AND ACHIEVEMENT**
Sex/Age		
Big Five [31]	Conceptual level	Psychological Wellbeing
Affect +/- [33]		
SR Short[4], S/L term[65], Self-Control[66]		
SR-NR-DR:	Study focus [1]	Psychological Wellbeing [54]
ED [49] (IATLP [9], SETLQ [59])	Learning Styles (ILS)[53]	Health [46]
CLINICAL [48]		Flourishing [45]
HEALTH [50]		
ORGAN [67]		
ICT [55]	Procedural level	Psychological stress
	Oral exposition skills[18]	Stress and Anxiety Responses [36]
	Written exam skills[25]	
	Metacognitive skills: Study and notetaking strategies[16, 19]	
	IATLP [9]	
	Metabehavioural skills: SR-short[4], Self-control[66]	
	Meta-affective skills: Coping[7, 34]	
	Meta-motivational skills: engagement-burnout[10,] engagement[39,38,37]	
	Meta-motivational study skills[22]	
	Difficulties of Emotional Regulation (DERS-16)[57]	
	Self-regulatory fatigue[63], EF[60]	Academic achievement
	Procrastination[44]	C, P, A (Spanish)[11]
		C, P, A (English)[12]
	Attitudinal level	
	Adaptability[62, 64]	
	Positivity[32], Resilience[3]	
	Psychological strengths[47], Spirituality[61], Self-compassion[70]	
	Achievement emotions[41,42,43], Test anxiety[8], Academic confidence[6]	
	Psychological Reactance[56]	

I	Emotional Reactivity [52]<br>Perfectionism[51]<br>Action-emotion[5], Impulsivity[68]	
**<u>CONTEXTUAL</u>**	**<u>CONTEXTUAL</u>**	**<u>CONTEXTUAL</u>**
· ER-ENR-ER:<br>ED [49]. FAM SUPP[58]<br>CLINICAL [48]<br>HEALTH [50]<br>ORGAN [67]<br>ICT [55]	SETLQ [59], IATLP [9]<br>ACAD STRESS ENS.[35]	IATLP [9]

GENERAL CONCEPTUAL MODEL OF COMPETENCE IN THE MANAGEMENT OF ACADEMIC STRESS AND PSYCHOLOGICAL WELLBEING IN EDUCATIONAL PSYCHOLOGY CONTEXT (de la Fuente, & Martínez-Vicente, 2022; IRR. No 00765-01162097)

TYPE OF SUBJECT: <u>UNIVERSITY</u>

**PRESAGE V.** →	**PROCESS V. (mediators)** →	**PRODUCT V.**
**<u>PERSONAL</u>**	**<u>PERSONAL</u>**	**<u>PERSONAL</u>**
**INDIVIDUAL**	**COMPETENCE IN MANAGEMENT STRESS AND WELLBEING**	**WELLBEING AND ACHIEVEMENT**
**Sex/Age**<br>**Big Five** [31]<br>Affect +/- [33]<br>SR Short[4], S/L term[65], Self-Control[66]<br>**SR-NR-DR**:<br>ED [49] (IATLP [9], SETLQ [59])<br>CLINICAL [48]<br>HEALTH [50]<br>ORGAN [67]<br>**ICT** [55]	<u>Conceptual level</u><br><br>**Study focus** [1]<br>Learning Styles (ILS)[53]<br><br><u>Procedural level</u><br><br>Oral exposition skills[18]<br>Written exam skills[25]<br>**Metacognitive skills: Study and notetaking strategies**[16, 19]<br>, **IATLP** [9]	<u>Psychological Wellbeing</u><br><br>**Psychological Wellbeing** [54]<br>**Health** [46]<br>**Flourishing** [45]<br><br><u>Psychological stress</u><br><br>**Stress and Anxiety Responses** [36]

	**Metabehavioural skills: SR-short[4], Self-control[66]**	
	**Meta-affective skills: coping**[7, 34]	
	**Meta-motivational skills**: **engagement-burnout[10,]** engagement[39,38,37]	
	**Meta-motivational study skills[22]**	
	Difficulties of Emotional Regulation (DERS-16)[57]	
	**Self-regulatory fatigue**[63], EF[60]	<u>Academic achievement</u>
	Procrastination[44]	**C, P, A (Spanish)**[11]
		**C, P, A (English)**[12]
	<u>Attitudinal level</u>	
	**Adaptability[62, 64]**	
	Positivity[32], **Resilience[3]**	
	**Psychological strengths[47]**, Spirituality[61], Self-compassion[70]	
	**Achievement emotions[41,42,43], Test anxiety[8], Academic confidence[6]**	
	Psychological Reactance[56]	
	Emotional Reactivity [52]	
	**Perfectionism[51]**	
	Action-emotion[5], Impulsivity[68]	
**<u>CONTEXTUAL</u>**	**<u>CONTEXTUAL</u>**	**<u>CONTEXTUAL</u>**
**ER-ENR-ER:**		
**ED** [49] **FAM SUPP** [58], **IATLP** [9]	**SETLQ**[59], **IATLP** [9]	**IATLP** [9]
CLINICAL [48]	**ACAD STRESS TEACH** [35]	
HEALTH [50]		
ORGAN [67]		
**ICT** [55]		

Note. In bold the variables selected for this psychological context

ANNEX 2. LIST OF INVENTORIES IN THE UTILITY MODEL (SELECTED: EDUCATIONAL PSYCHOLOGY AREA)

**AREA**	**EDUCATIONAL PSYCHOLOGY**	**(FOR UNIVERSITY STUDENTS OR SIMILAR)**	
**NUM. INVENT.**	**1) PRESAGE VARIABLES**		
	**1.1. Individuals**		
31	BFQ-N	Big Five Questionnaire	Cuestionario de los Cinco Grandes
49	SRL-ERL. ACADEMIC LEARNING	Self- vs External- Regulation Scale in Behavior Academic Learning	Escala de Auto-Regulación vs Hetero-Regulación Com...
55	SR vs ER. TICS	Self- vs External- Regulation Scale for Assessment...	Escala para la Evaluación de la Auto-Regulación vs...
	**1.2. Contextuals**		
49	SRL-ERL. ACADEMIC LEARNING	Self- vs External- Regulation Scale in Behavior Academic Learning	Escala de Auto-Regulación vs Hetero-Regulación Com...
55	SR vs ER. TICS	Self- vs External- Regulation Scale for Assessment...	Escala para la Evaluación de la Auto-Regulación vs...
58	AliYPE	Adult Involvement in Young People's Education	Participación de los adultos en la educación de los jóvenes
	**2) PROCESS VARIABLES**		
	**2.1. Individuals**		
	**Conceptuals**		
1	R-SPQ-2F-UNI	Revised Study Process Questionnaire (UNI)	Proceso de Estudio Revisado (UNI)
	**Procedurals**		
9	EIPEA/ IATLP- Abbreviated	IATLP Scale - Abbreviated	EIEPEA /Evaluación Interactiva del Proceso de Enseñanza-Aprendizaje.
4	SRQ-Abbreviated	Self-Regulation Questionnaire Abbreviated	Cuestionario de Autorregulación Abreviado
7	EEC-UNI	Questionnaire on Coping Strategies (UNI)	Cuestionario de Estrategias de Afrontamiento (UNI)
44	PASS	Procrastination Asessment Scale- Students	Escala de Evaluación de la Procrastinación
	**Attitudinals**		
3	CD-RISC-UNI	ResilienceScale (UNI)	Escala de Resiliencia (UNI)
47	VIA	VIA Survey of Character Strengths	Cuestionario VIA de Fortalezas Personales
41	AEQ-C-RE	Achievement Emotions Questionnaire: Class-Related ...	Cuestionario de Emociones de Logro: Emociones Rela...
42	AEQ-L-RE	Achievement Emotions Questionnaire: Learning-Relat...	Cuestionario de Emociones de Logro: Emociones Rela...
43	AEQ-TE	Achievement Emotions Questionnaire: Test Emotions	Cuestionario de Emociones de Logro: Emociones ante...
6	ABC-UNI	Academic Behavioural Confidence Scale (UNI)	Escala de Confianza Académica (UNI)
	**2.2. Contextuals**		
9	EIPEA/ IATLP- Abbreviated	IATLP Scale - Abbreviated	EIEPEA /Evaluación Interactiva del Proceso de Enseñanza-Aprendizaje.
	**3) PRODUCT VARIABLES**		
	**3.1 Individuals**		
	**Psychological Well-Being**		

45	FSI	Flourishing Scale Inventory	Cuestionario de Prosperidad Personal
46	AHI	Academic Health Inventory	Cuestionario de Salud Académica
54	PWB-S	Ryff Psychological Well-Being Scale	Escalas de Bienestar Psicológico Ryff
	**Academic Stress**		
36	R-CEA	Stress and anxiety response	Respuesta de estrés y ansiedad
	**Academic Achievement**		
11	Achiv-SPA	Achievement Scale (Only for Spanish-speaking Stude...	Cuestionario de Rendimiento (Solo para alumnos esp...
12	Achiv-ENG	Achievement Questionnaire (Only for English-speaki...	Cuestionario de Rendimiento (Sólo para alumnos Ing...
	**TOTAL: 20 INVENTORIES**		
